# Unexpected Seed Migration in Prostate Brachytherapy Implants Coincident with Change in Seed Stranding Product

**DOI:** 10.7759/cureus.1243

**Published:** 2017-05-12

**Authors:** Jim Rose, Derek Liu, Oleksandr Boychak, Ron Sloboda, Nadeem Pervez, Albert Murtha, Don Yee, John Amanie, Nawaid Usmani

**Affiliations:** 1 Radiation Oncology, BC Cancer Agency – Abbotsford Centre; 2 Radiation Oncology, Cross Cancer Institute, University of Alberta; 3 Department of Oncology, University of Alberta

**Keywords:** prostate brachytherapy, low dose rate, quality assurance

## Abstract

Purpose: This study was undertaken to determine if significant seed migration occurred when our institution changed seed products by comparing patterns of seed migration in implants containing different stranding material.

Methods and Materials: Day 0 and Day 30 CT scans were registered by the contoured prostate center of mass. An implant reconstruction program identified seeds on CT according to the pre-plan, enabling one-to-one correspondence between Day 0 and Day 30 seeds. Significant seed migration was defined by review of seeds that migrated > 2 cm outside the prostate or appearance in unexpected locations. Results: Twenty-five (149, 16.8%) new strands displayed movement > 2 cm between Day 0 and Day 30 compared with just 2/118 (1.7%) of the standard strands. Six out of 26 (23%) patients with new strands displayed significant migration compared with 2/13 (14%) of patients with standard strands. In the six patients with new strands and significant migration, a mean of four strands (17%, range: 2-8 per patient) migrated significantly with 65% due to whole strand migration, 25% due to strand breakage, and 10% strand clumping. In the control group, only two strands (2%) migrated significantly, both due to strand breakage. Despite the greater seed movement with the new strands, Day 0 and Day 30 dosimetry was acceptable.

Conclusion: In this short report, we identified that a change to a new strand type was associated with unexpected significant seed movement compared to our typical strands. Since seed movement can arise from unexpected causes, it is important to maintain quality assurance practices when a change in technique or infrastructure is instituted.

## Introduction

Low dose rate (LDR) permanent seed prostate brachytherapy is an established curative treatment for men with favorable-risk prostate cancer. The success of treatment depends on patient risk factors and implant quality, the latter that is assessed with postoperative dosimetry involving computed tomography (CT) and magnetic resonance imaging (MRI) up to 30 days post-implant [1-3]. Postoperative seed migration either locally, through the venous system, in urine, or during ejaculation may lead to suboptimal dosimetry and toxicity [4-6]. Factors that may influence seed migration include the use of stranded vs loose seeds, planning algorithm, the length of the seed train, use of sagittal imaging, urethral contrast, and others [6-8].

In the course of post-implant quality assurance of patients treated with permanent seed brachytherapy at our institution, we encountered a series of patients in which some had qualitatively significant seed migration beyond what we had previously encountered. Strands often appeared to be clumped within the prostate or were noticed in bizarre locations, such as the ischiorectal fossa. This series of patients was treated after the transition to a new seed stranding material. In this report, we describe our experience with sudden, unexpected strand migration corresponding to the time when we changed our seed products.

## Materials and methods

Our institution performs between 100 and 150 LDR prostate brachytherapy implants each year between five radiation oncologists. Between May and September 2013, 40 patients with favorable prostate cancer (T1c-T2b, Gleason 6-7, PSA < 20) were treated with transperineal LDR Iodine-125 (^125^I) permanent prostate brachytherapy. All patients underwent transrectal ultrasound (TRUS) and transverse images were collected every 5 mm for pre-planning using a BK Pro Focus (model 2202, 6 MHz) (Analogic Corp., Peabody, MA). Images were imported into the VariSeed™ planning system (Varian Medical Systems, Palo Alto, CA) and the prostate clinical target volume (CTV) and urethra were contoured by the radiation oncologist. A planning target volume (PTV) margin of 3 mm laterally and anteriorly, 5 mm inferiorly, and 0 mm superiorly and posteriorly were applied. A non-uniform distribution of seeds was placed to provide a minimum peripheral dose (MPD) of 145 Gy. The urethral dose was limited to < 150% of MPD. Our standard ^125^I seeds (Oncura model 6711) (GE Healthcare) were provided in pre-loaded needles by Biocompatibles, Inc. (Ottawa, ON, Canada) and were custom-stranded according to the pre-plan. Standard strands consisted of a braided carrier of 90/10 glycolide/L-lactide with < 90-day absorption. Leading and trailing half spacers were present. A description of our planning algorithm has been described previously [9]. Source strength was 0.395 mCi per seed. Up to five additional unplanned seeds were available intraoperatively to correct for dose distribution deficiencies.

Implants were done by five experienced radiation oncologists, including implants performed by a brachytherapy fellow close to the completion of his/her training under the oncologist’s supervision. Implants were done in the operating room under general anesthetic and with sterile technique. An ultrasound probe was inserted into the rectum and the prostate was aligned to reproduce the image set from the pre-plan TRUS. Using a standard template, pre-loaded needles were inserted into the prostate starting at the anterior row, one needle at a time. Needle depth was confirmed on axial and sagittal TRUS images before seeds were manually deposited before moving to the next needle. At the conclusion of the implant, a Foley catheter was placed. Patients underwent Day 0 quality assurance CT scanning after which the Foley catheter was removed. The intraprostatic urethra and peri-prostatic outer rectal wall were contoured. For data analysis, all contours were done by a single observer. No patients received supplemental external beam radiotherapy.

In early 2013, the stranding material changed and 26 patients were implanted with the new strands. The new strands were composed of a non-braided clear monofilament of 20/80 glycolide/L-lactide with 140-180 day absorption. No leading spacers were included, and information from the supplier indicates that these strands demonstrate less flexibility than our typical strands. While using this new product, routine quality assurance revealed qualitatively significant migration of strands in six of the 26 patients, often to bizarre locations, such as the ischiorectal fossa. This migration was particularly unusual for our institution, as we have previous experience in patients with serial imaging and no such significant migration [9]. We initiated an additional Day 30 CT scan in these patients to analyze seed fixity. With these findings, we reverted back to our standard stranding product. These six patients, as well as a cohort of five subsequent patients implanted after we resumed with standard strands, were analyzed.

**Figure 1:**
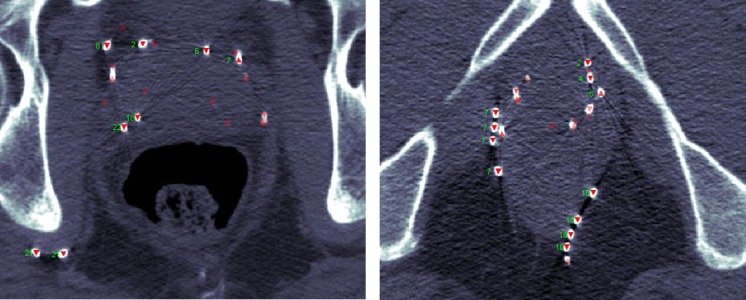
Day 30 axial computed tomographic images showing seed migration to unexpected locations

In these 11 patients, strand migration between Day 0 and Day 30 quality assurance CT scans was quantified and spatially registered by the prostate center of mass. An implant reconstruction program developed in-house at the British Columbia Cancer Agency was used to uniquely identify seeds on the CT according to the pre-plan loading diagram, enabling one-to-one correspondence between Day 0 and Day 30 seeds [10]. A subset of Day 0 seeds required manual identification, which was aided by a comparison with the loading diagram. An analysis was performed using in-house software, MATLAB® (The Mathworks, Natick, MA, USA). Significant seed migration was defined qualitatively by review of seeds that migrated > 2 cm outside the prostate contour, seeds that were lost, or seeds that appeared in unexpected locations, such as the ischiorectal fossa (Figure 1). Patient demographic information was collected and a detailed analysis of Day 0 and Day 30 seed migration was undertaken.

## Results

A series of 26 consecutive patients were implanted with the new strand material. Upon review of the postoperative quality assurance CT, six patients (23%) were found to have > 1 strands with migration > 2 cm beyond the prostate. Of these 26 patients, 25/605 (4.1%) of implanted strands migrated > 2 cm. After returning to our standard strands, a further 14 consecutive patients were implanted with only two patients showing the migration of a single strand > 2 cm beyond the prostate (2/340 (0.6%) strands implanted).

These six patients implanted with the new strands that displayed significant strand migration were compared with a series of five patients matched with similar operative characteristics (i.e., same implanting oncologist) implanted after we returned to the standard stranding product. Table 1 compares the demographic and treatment-related information of these two groups.

**Table 1:** Characteristics of Patients Implanted with New Strands and Standard Strands n: number of patients; PSA: prostate specific antigen; ADT: androgen deprivation therapy; TRUS: transrectal ultrasound; cc: cubic centimeters; CT: computed tomography

		New strands (n = 6)	Standard strands (n = 5)
Age, median (range in years)		63 (50.4 - 64.5)	67.3 (53 - 67.6)
Clinical stage (n)			
	T1c	3	1
	T2a	2	1
	T2b	1	2
	T2c	0	1
Gleason score			
	3,3	4	1
	3,4	2	3
	4,3	0	1
Initial PSA, median (range), ng/ml		7.1 (3.8 - 8.2)	8.4 (5.2 - 9.4)
Neoadjuvant +/- Concurrent ADT (n)		0	1
Preop TRUS volume, median (range), cc		57.4 (37.4 - 63.1)	41.9 (33.3 - 58.6)
Number of seeds implanted, median (range)		116 (95 - 125)	97 (84 - 123)
Number of needles, median (range)		25 (22 - 27)	23 (20 - 29)
Day 0 TRUS volume, median (range), cc		60.6 (46.6 - 63)	41.4 (34.6 - 57.4)
Day 30 CT volume, median (range), cc		44.8 (30.8 - 53.8)	36.5 (33.3 - 55.52)

Significant strand migration (i.e., > 2 cm) was classified as occurring due to strand breakage (where a partial strand is present), whole strand migration, or clumping and occurred with a greater frequency in patients implanted with new strands (Table 2). In patients implanted with the new strands, 25/149 strands (16.8%) displayed movement > 2 cm between Day 0 and Day 30 compared with just 2/118 strands (1.7%) in those implanted with the standard strands. In patients implanted with the new strands, out of the strands showing significant migration, 7/25 (28%) were planned as short strands containing 2-3 seeds. All of these short strands showed whole strand migration rather than breakage or clumping, and 6/7 (86%) of these strands were implanted entirely outside of the prostate. Sixteen of 25 (64%) of the new strands showing significant migration contained 5-7 seeds per strand. Half of these strands (8/16) exhibited breakage and migration of the smaller strand fragment. Thus, strands affected by significant migration tended to be either short strands (i.e., 2-3 seeds) implanted outside the prostate or long strands (i.e., 5-7 seeds) with breakage and migration of a fragment.

**Table 2:** Description of Strand Migration with New Strands and Standard Strands n: number of strands/seeds; cm: centimeters

							Migration due to:								
	Patient		Strands implanted (n)		Strands with > 2 cm migration (n, % of total)		Breakage (n)	Whole strand migration (n)		Clumping (n)	Seeds lost (n)				
New strands																				
	A		27		8 (29.6)		4		4	0		5			
	B		22		3 (13.6)		0		2	1		5			
	C		26		4 (15.4)		2		1	1		0			
	D		24		2 (8.3)		0		2	0		0			
	E		24		3 (12.5)		1		2	0		0			
	F		26		5 (19.2)		1		4	0		3			
Total (%)			149		25 (16.8)		8 (5.4)		15 (10.1)	2 (1.3)		13 (1.8)			
Standard strands															
	A		22		0		0		0	0		0			
	B		20		1 (5)		1		0	0		2			
	C		29		0		0		0	0		0			
	D		24		1 (5)		1		0	0		1			
	E		23		0		0		0	0		0			
Total (%)			118		2 (1.7)		2 (1.7)		0	0		3 (0.6)			

**Figure 2:**
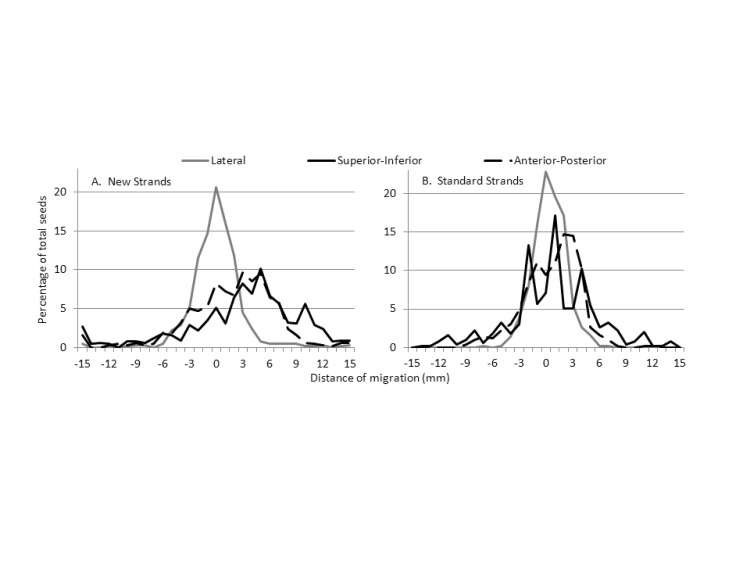
Absolute seed movement in three dimensions between Day 0 and Day 30 computed tomography A) New strands (n = 682 seeds); B) Typical strands (n = 490 seeds)

Individual seed movement was also compared between Day 0 and Day 30 in the six patients implanted with the new strands who had significant strand migration and the subsequent group of five patients implanted with standard strands. Figure 2 shows that seed movement of 0 - 5 mm tended to occur at a greater frequency for standard strands but movement > 5 mm occurred more frequently with the new strands. The new strands exhibited a systematic migration of seeds (median ± standard deviation) relative to the prostate contour in the superior (3.7 ± 8.8 mm) and anterior (2.1 ± 7.8 mm) directions but not in the lateral direction (-0.4 ± 3.6 mm). Seed migration with standard strands was 0.8 ± 5.0 mm, 0.7 ± 3.1 mm, and -0.1 ± 1.8 mm, respectively.

Despite more frequent and greater strand migration with the new strands compared to standard strands, Day 30 D90 and V100 were adequate with implants, including the new stranding material generally being colder (Table 3). Reimplantation was discussed but was not performed on any patients, and no patient received supplementary androgen deprivation therapy (ADT). With the exception of one patient implanted with standard strands, Day 0 rectal volume receiving > 100% of prescription dose (RV100) was within tolerance for both groups. In the group implanted with new strands and having significant strand migration, 3/6 patients had negligible changes in RV100 by Day 30 (range: -0.25 – 0.17). One patient’s RV 100 increased from 0.08 to 3.45 cc and another’s increased from 0.17 to 2 cc (data from one patient missing). Likewise, in the group implanted with standard strands, negligible changes were seen in 3/4 patients (range: -0.23 – 0.44 cc), whereas RV100 increased from 0.79 to 2.57 cc in one patient (data from one patient missing). Urethral volume receiving > 150% of prescription dose (UV150) data was not available. No patients experienced Grade 3/4 toxicity.

**Table 3:** Day 0 and Day 30 Dosimetry for New Strands and Standard Strands D90: dose received by > 90% of target; Gy: Gray; V100 (V150, V200) – target volume receiving > 100%, 150%, and 200% of prescription dose; RV100: rectal volume receiving > 100% of prescription dose; cc: cubic centimeters

		New strands (n = 6)	Standard strands (n = 5)	
Day 0 CT	D90 (Gy)	152.5 (139 – 170.6)	146 (125.9 – 180.1)	
	V100 (%)	92.7 (84.7 – 98.2)	90.3 (84.1 – 97.4)	
	V150 (%)	53.2 (34.7 – 61.1)	47 (46 – 73.3)	
	V200 (%)	21.1 (14.5 – 23)	20.6 (16.3 – 41.1)	
	RV100 (cc)	0.13 (0 – 0.55)	0.79 (0 – 1.58)	
Day 30 CT	D90 (Gy)	156.8 (131.6 – 175)	166.3 (152.6 – 184.9)	
	V100 (%)	92.7 (78 – 98.4)	94.9 (92.4 – 98.3)	
	V150 (%)	54.5 (31.2 – 65.3)	70.1 (58.22 – 71)	
	V200 (%)	19.4 (12.7 – 29.5)	36.2 (32.1 – 38.6)	
	RV100 (cc)	0.47 (0.07 – 3.45)	1.09 (0.46 – 2.57)	
Median percent change (range) at Day 30 relative to Day 0				
	D90	-3.5 (-10.5 – 11.9)	8.1 (-7.7 – 27)	
	V100	-2.7 (-6.7 – 4.1)	3.1 (-2.6 – 9.8)	
	V150	-2.3 (-10.2 – 14.9)	24.3 (-4.4 – 54.3)	
	V200	-2.1 (-12.4 – 33)	55.9 (-11.9 – 137.5)	
	RV100	56.7 (-45.5 – 4212)	110.8 (-25.8 – 2200)	

## Discussion

This short report of a series of patients treated with LDR prostate brachytherapy shows greater strand migration in a subset of patients treated with the same implant technique but a different stranding material. Two major differences were observed in the pattern of migration between the new strands and the typical strands. Firstly, among new strands, there was a greater degree of migration of seeds in the superior and anterior direction compared with standard strands (Figure 2). In addition, a significant migration of strands (i.e., > 2 cm) was a more frequent occurrence with the new strands (Table 2). Although Day 30 D90 and V100 were generally acceptable, implants that displayed significant seed migration with the new stranding material were generally cooler (Table 3). The degree and magnitude of strand movement observed could have resulted in under-dosage in a larger sample size or longer follow-up. It is difficult to appreciate the full dosimetric impact of this migration, given the static assessment of dosimetry at a few selected time-points, with the possibility of additional migration not assessed after Day 30. This report shows that seed migration can arise from unexpected or unknown causes and thorough quality assurance must be maintained with any change in procedure or implant materials.

Our appreciation that a clinically significant extent of seed migration was occurring with this new product stems from our previous experience in studying seed migration [9]. Although this previous experience was from a different institution, the products and techniques were similar to our current methods and included one common physician. The identification of 16.8% of strands with the new product migrating greater than 2 cm was in stark contrast to our previous publication of no strands from 233 strands implanted migrating more than 1.4 cm. This prior work also showed that strands were moving less than 1 mm from Day 0 to Day 30, supporting our conclusion that there typically is no significant pattern of migration of seeds from Day 0 to Day 30. This background allowed us to appreciate that something different was occurring in patients implanted with the new strands.

Our implant technique remained consistent throughout this series of patients, and we hypothesize that the increased strand migration was a consequence of the change in stranding material. Unlike our standard strands, the new strands did not have leading spacers and were less flexible. Some authors have speculated that leading spacers may influence strand migration by providing a means of penetration through needle tracts [11], although migration was less if leading spacers were present in our series.

It is also possible that strand flexibility played a larger role in strand migration. A less flexible strand may be more susceptible to migration due to prostate deformation, resolution of edema, and muscular contraction. This is supported by the observation in our series with a greater extent of migration of seeds in the superior and anterior direction compared with standard strands (Figure 2). Early postoperative edema of the genitourinary diaphragm tends to push the prostate superiorly, and as edema resolves by Day 30, the prostate moves inferiorly [11-12]. In addition, Day 0 intraprostatic edema is greatest in the superior and anterior directions, resolving by Day 30 [13]. A less flexible strand may gain traction in extraprostatic tissues and may be less likely to move with the prostate as edema resolves. In this series, Day 0 prostate edema was greater for patients implanted with the new strands (135%) compared to standard strands (113%), which may have promoted strand migration (Table 1).

In this small case series, implants done with the new strands were more likely to show significant migration (i.e., > 2 cm) sometimes to unexpected locations, such as the ischiorectal fossa (Figure 1). Migration of this magnitude is not likely the result of resolving edema alone. Intraprostatic edema on Day 0 as measured on MRI is approximately 10% in the superior and anterior directions [13], and excursion of the genitourinary diaphragm as edema resolves from Day 0 to Day 30 has been estimated at 4 + 3.5 mm [11]. Strand breakage occurred more frequently with the new strands, which may explain some instances of significant migration (Table 2). We speculate that a combination of less strand flexibility and muscular contraction or loss through blood vessels may have played a role. 

These observations have led to a number of changes to our practice. We are no longer using the less flexible strands and have returned to our standard product (see Materials and Methods). We have also modified our planning approach to minimize extra-prostatic strands as our qualitative observations showed that significant migration tended to occur in entirely extra-prostatic strands. These modifications have greatly reduced significant strand migration. Any future changes in practice will undergo strict quality assurance.

This study has several limitations. This is a small, non-randomized case series showing a greater frequency of movement in a subset of, but not all, patients implanted with a different stranding material. One of the greatest limitations of this study is the arbitrary manner of selecting patients for analysis. After identifying a series of consecutive patients with significant migration using the new product, we focused on a cohort of 14 consecutive patients with other products as a second cohort for comparison. Although this strategy for selecting patients was not ideal, it provided a framework for easily comparing patient populations. As there are many patient and technique factors that influence strand migration, we cannot conclude with certainty that the stranding material was responsible for the strand migration. The purpose of this report, however, is to describe our experience with sudden unexpected strand migration and the impact of this on our practice. The accuracy of contouring and dosimetry could have been improved by using postoperative MRI and CT scans [14]. Ideally, patients implanted with the new strands should have been compared to patients implanted with the standard strands before the switch was made as knowledge of the recent seed migration may have introduced subtle changes in technique. Patients implanted before the switch to the new stranding product did not have Day 30 CT scans and could not be compared to patients implanted with the new strands. Lastly, we cannot determine the full dosimetric impact of this migration as there may have been further strand migration after Day 30.

## Conclusions

This report describes a series of LDR prostate seed implants that displayed significant and unexpected strand migration that coincided with a change in stranding material. The small sample size analyzed in this study prevents definitive conclusions about seed migration but provides insight about the importance of quality assurance measures when any changes in products are implemented. We have since modified our practice and will institute stricter quality assurance for any future changes in practice.
